# Clinical Study in 11 Cases of Endobronchial Foreign Body

**DOI:** 10.1155/DTE.2.197

**Published:** 1996

**Authors:** Shinji Atagi, Kiyoyuki Furuse, Masaaki Kawahara, Nagahisa Kodama, Mitsumasa Ogawara, Tatsuya Okada, Yuji Kawaguchi, Takao Kamimori, Mitsunobu Nakao, Nobuyuki Naka

**Affiliations:** Department of Internal Medicine National Kinki Central Hospital for Chest Diseases 1180 Nagasone Osaka Sakai 591 Japan

## Abstract

We report 11 cases of endobronchial foreign body. From January 1982 through December 1994, a total of 11 cases were diagnosed roentogenographically and bronchoscopically at our hospital. These patients consisted of 10 men and 1 woman with a mean age of 58.5 years (range 33 to 77 years). Symptoms on presenting were usually cough, sputum, or chest pain. The foreign bodies were inorganic in 10 cases and of organic origin in 1 case. Three patients were not aware that they had aspirated a foreign body. In 9 patients, the endobronchial foreign bodies were successfully removed endoscopically. One patient spontaneously expectorated the foreign body before bronchoscopy. One patient underwent thoracotomy because the foreign body could not be removed bronchoscopically. There were no severe complications during or after the endoscopic removal of the foreign bodies, but in one patient extraction of the foreign body caused pneumonia after bronchoscopy. In conclusion, flexible bronchoscopy is useful for the diagnosis and treatment of endobronchial foreign bodies.


*Diagnostic and Therapeutic Endoscopy*, 1996, Vol. 2, pp. 197–202
Reprints available directly from the publisher
Photocopying permitted by license only

© 1996 OPA (Overseas Publishers Association)
Amsterdam B. V. Published in The Netherlands
by Harwood Academic Publishers GmbH
Printed in Singapore

### Clinical Study in 11 Cases of Endobronchial Foreign Body

SHINJI ATAGI, KIYOYUKI FURUSE, MASAAKI KAWAHARA, NAGAHISA KODAMA, MITSUMASA OGAWARA, TATSUYA OKADA, YUJI KAWAGUCHI, TAKAO KAMIMORI, MITSUNOBU NAKAO, and NOBUYUKI NAKA

*Department of Internal Medicine, National Kinki Central Hospital for Chest Diseases, Osaka, Japan*

*(Received May 22, 1995; in final form July 10, 1995)*

We report 11 cases of endobronchial foreign body. From January 1982 through December 1994, a total of 11 cases were diagnosed roentogenographically and bronchoscopically at our hospital. These patients consisted of 10 men and 1 woman with a mean age of 58.5 years (range 33 to 77 years). Symptoms on presenting were usually cough, sputum, or chest pain. The foreign bodies were inorganic in 10 cases and of organic origin in 1 case. Three patients were not aware that they had aspirated a foreign body. In 9 patients, the endobronchial foreign bodies were successfully removed endoscopically. One patient spontaneously expectorated the foreign body before bronchoscopy. One patient underwent thoracotomy because the foreign body could not be removed bronchoscopically. There were no severe complications during or after the endoscopic removal of the foreign bodies, but in one patient extraction of the foreign body caused pneumonia after bronchoscopy. In conclusion, flexible bronchoscopy is useful for the diagnosis and treatment of endobronchial foreign bodies.

**KEY WORDS:** Bronchoscopy, endobronchial foreign body

### INTRODUCTION

Flexible bronchoscopy is useful not only in the diagnosis and treatment of pulmonary diseases, but also in the management of endobronchial foreign bodies. The presence of endobronchial foreign bodies is comparatively rare in adults (1). Symptoms on aspiration of foreign bodies usually include severe cough, dyspnea, and wheezing. In a worst case scenario, they can cause death due to suffocation. Adult patients usually notice that they have aspirated foreign bodies, and the diagnosis is then easy. If the patients are not aware that they have aspirated a foreign body, there may be a misdiagnosis of bronchial asthma or chronic bronchitis. In patients with longstanding endobronchial foreign bodies, complications may include secondary infection, atelectasis, and bronchiectasis (2,3). In this study, we retrospectively investigated cases of endobronchial foreign body in our hospital.

Address for correspondence: Dr. Shinji Atagi, the Department of Internal Medicine, National Kinki Central Hospital for Chest Diseases, 1180 Nagasone, Sakai, Osaka, Japan 591.

### PATIENTS AND METHODS

We reviewed the medical records of 11 patients referred to the National Kinki Central Hospital for Chest Diseases for whom a diagnosis of endobronchial foreign body was made during January 1982 and December 1994.

### RESULTS

Characteristics of the 11 patients are summarized in Table 1. These patients were 10 men and 1 woman with the mean age of 58.5 years (range, 33 to 77 years). About one-half of the incidents occurred when patients were in their 60s. Symptoms at the time of the first visit to our hospital were usually cough, sputum, or chest pain. Seven patients aspirated a foreign body during dental treatment, one patient while working at carpentry, one patient while sleeping, and one patient during an epileptic fit.

Three of the 11 patients (27.3%) were not aware that they had aspirated a foreign body. Case 7 was a 70-year-old man who had aspirated an artificial denture during dental treatment, although he thought he had swallowed it. Case 10 was a 54-year-old man who suddenly became unconscious due to an epileptic fit and fell to the ground. A fellow worker saw him unconsciously biting a stone on the ground. Case 11 was a 53-year-old man who aspirated a fish bone. The patient did not realize he had aspirated the fish bone, and he had no history of becoming unconscious for any reason.

The plain chest radiographs of 10 patients (91%) showed a definite foreign body shadow. All of them were of inorganic origin, mostly artificial dentures. The others were a nail, a root canal reamer, and a stone. One foreign body (Case 11) was organic, the fish bone, and the plain chest radiograph showed the atelectasis of the right lower lobe with no evidence of a foreign body. Table 2 demonstrates the site of the foreign body within the airways of the patients as verified by plain chest radiograph and/or bronchoscopy. There were no foreign bodies in the trachea. The right bronchial tree was more often affected (63.6%) than the left (36.4%). No patient aspirated more than one foreign body.

Bronchoscopic findings in patients with longstanding metallic material and stone in the bronchus (Figs. 1 to 3) showed redness, edema, and granulation of bronchial mucosa bronchoscopically. In the patient with the fish bone aspiration (Fig. 4), the mucosa of the right lower lobe bronchus was narrowed by granulation. One month later, the granulomatous stenosis improved, but the chest radiographic findings remained slightly atelectasis. In 9 patients, the foreign body was removed successfully by bronchoscopy under local anesthesia. In one patient (Case 3), the foreign body was lodged in the right $B^{10}$, necessitating removal by thoracotomy without pulmonary resection. One patient (Case 7) coughed out the foreign body spontaneously before bronchoscopy. It had been in the bronchus for 7 months. In 5 patients (45.5%), the foreign bodies were removed within the first 48 hours after the aspiration. In these cases, the patients or dentists themselves either specifically reported the event or had a reasonable suspicion that an aspiration had taken place. In 5 patients (45.5%), they were removed from 23 days to 10 months later. The reason for delay of treatment was that all these patients had already developed severe symptoms. One patient did not notice when he had aspirated the foreign body, but he began to notice cough and sputum 7 months before admission. There were no severe complications during or after the extracting maneuver. In one patient (Case 4) extraction of the artificial denture caused pneumonia after bronchoscopy.

**Table 1** Characteristics of 11 patients

| *Case* | *Age (years)* | *Sex* | *Chief Complaint* | *State of Event* | *History* | *Underlying Disease* |
|---|---|---|---|---|---|---|
| 1 | 44 | Male | Cough, fever | While at carpentry | + | – |
| 2 | 33 | Male | Chest pain | During dental treatment | + | – |
| 3 | 57 | Male | Cough, hemosputum, back pain | During dental treatment | + | – |
| 4 | 77 | Female | Cough, sputum | While sleeping | + | – |
| 5 | 53 | Male | Chest X-ray abnormality | During dental treatment | + | Apoplexy |
| 6 | 59 | Male | Chest X-ray abnormality | During dental treatment | + | – |
| 7 | 70 | Male | Cough | During dental treatment | – | Parkinson's disease |
| 8 | 69 | Male | Chest X-ray abnormality | During dental treatment | + | – |
| 9 | 75 | Male | Cough | During dental treatment | + | – |
| 10 | 54 | Male | Cough | At epileptic fit | – | Epilepsy |
| 11 | 53 | Male | Cough, hemosputum, chest pain | Unknown | – | – |

**Table 2** Foreign bodies found in 11 patients

| *Case* | *Kind of Foreign Body* | *Radiopacity* | *Location** | *Treatment/Term* | *Complication* |
|---|---|---|---|---|---|
| 1 | Nail | + | Rt upper lobe br | Endoscopically/2 months | – |
| 2 | Root canal reamer | + | Lt main br | Endoscopically/2 days | – |
| 3 | Artificial denture | + | Rt $B^{10}$ | Thoracotomy/10 months | – |
| 4 | Artificial denture | + | Rt basal br. | Endoscopically/40 days | Pneumonia |
| 5 | Artificial denture | + | Rt $B^4$ | Endoscopically/on the day | – |
| 6 | Artificial denture | + | Lt basal br | Endoscopically/on the day | – |
| 7 | Artificial denture | + | Rt basal br | Spontaneous expulsion/7 months | – |
| 8 | Artificial denture | + | Middle lobe br | Endoscopically/on the day | – |
| 9 | Artificial denture | + | Lt basal br | Endoscopically/on the day | – |
| 10 | Stone | + | Lt main br | Endoscopically/23 days | – |
| 11 | Fish bone | – | Rt lower lobe br | Endoscopically/unknown | – |

*Rt, right; lt, left; br, bronchus.

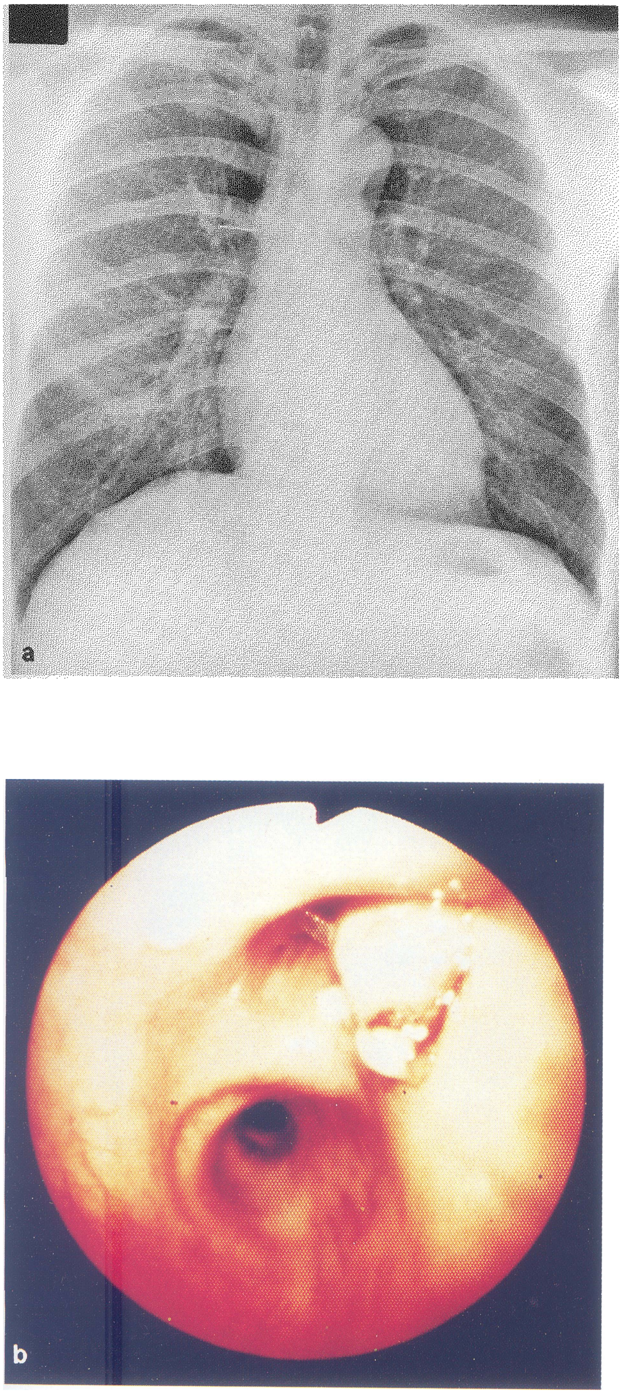

Figure 1 Case 1. A. Plain chest radiograph 2 months after aspiration of a nail shows the foreign body in the right upper lobe bronchus. B. A foreign body and granulomatus lesion can be seen in the right upper lobe bronchus.

### DISCUSSION

Eleven cases of endobronchial foreign body have been referred to our hospital during the last 13 years, from 1982 to 1994. Inhalation of foreign bodies is a common problem in children (4,5). Foreign body inhalation was 45 times more common in children than in adults and nearly 83% of patients were younger than 5 years of age.[1] Our hospital has no department of pediatrics, and all our patients were adults.

In this study, all but one patient were men. It has been proven that boys are more prone to develop this problem than girls (4–6). Gupta et al. (7) suggested that boys by nature are more curious and inquisitive than girls. However, the cause of male predominance in adults is unknown. In adults, this disease is more likely to occur among the elderly and those with underlying disease, such as dementia or stroke. In this study, three patients had underlying disease: apoplexy (Case 3), Parkinson's disease (Case 7), and epilepsy (Case 10), respectively.

In our study, eight patients (72.7%) had a clear history of foreign body inhalation. Several reports have shown that a history of inhalation in children were available in 38 to 85% of patients (5,8,9). A plain chest radiograph does not always contribute to the diagnosis; radiolucent bodies cannot be diagnosed on plain chest radiographs. A normal chest radiograph can be obtained in about one-fourth of patients.[4] The clinical picture of this disease depends upon the nature, size, and the site of lodgement of the foreign body in the airway. In children, most foreign bodies are of organic origin (such as nutty substances) (1,8). Longstanding endobronchial foreign bodies cause irritation or inflammation in the respiratory tract. Vegetables are more dangerous than other kinds of foreign bodies (metallic and plastic material), because the organic origin can cause both a clinical syndrome of mechanical obstruction and one due to either chemical irritation or allergic reaction as a result of local absorption of antigenic protein material (10). In this study, most of the foreign bodies (81.8%) were of metallic origin. In our patients with longstanding foreign bodies, bronchoscopy revealed inflammation and granulation around the foreign bodies. After these foreign bodies were removed, the inflammatory changes in the bronchus was usually resolved (11). In most of our patients, these foreign bodies were removed bronchoscopically. If a foreign body was buried in the bronchial wall by severe granulation, the patient may have been required to have cauterization by YAG laser or bronchotomy. It is difficult to remove foreign bodies such as spherical or fragile substances, bronchoscopically. In these cases, a Fogarty catheter or other types of forceps may be useful. Pulmonary resection may be required because of secondary abscess and bronchiectasis.

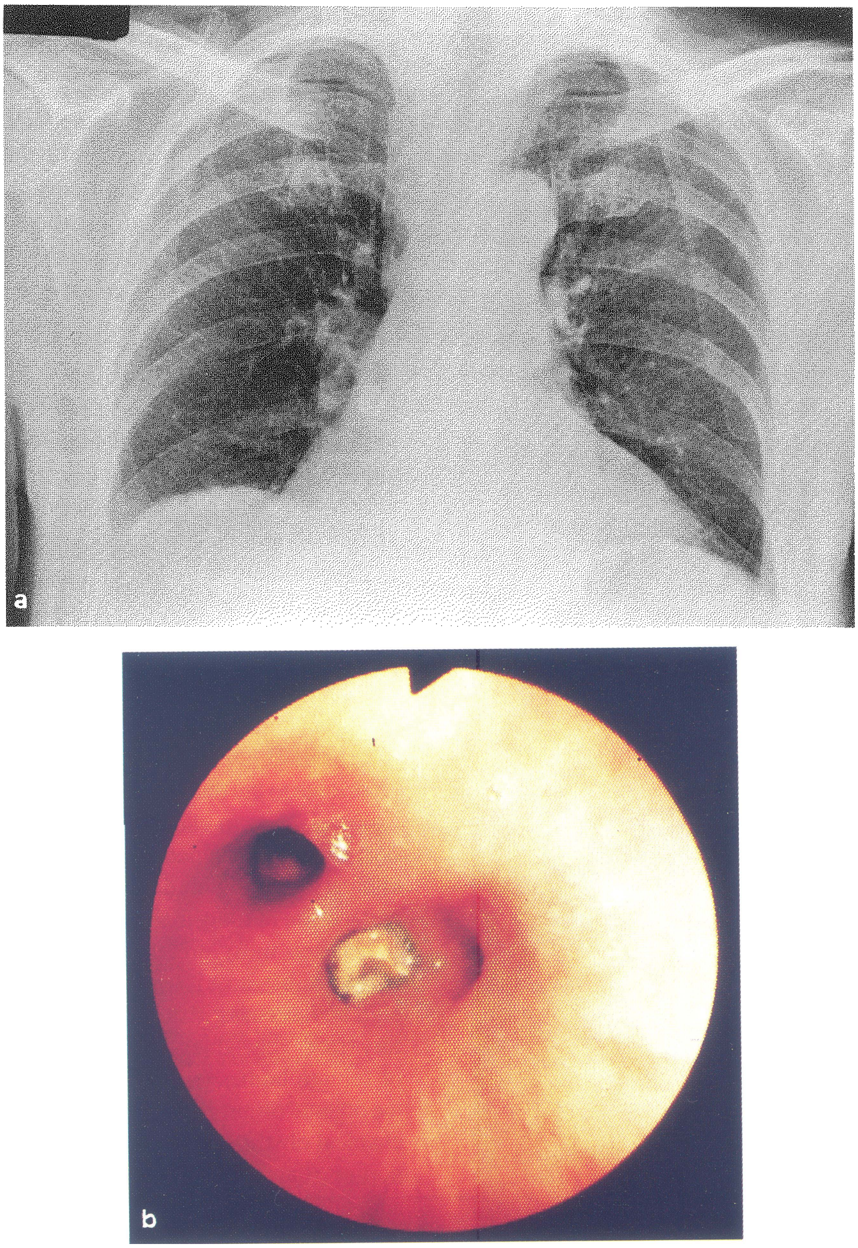

**Figure 2** Case 4. A. Plain chest radiograph 40 days after aspiration of the artificial denture shows the foreign body in the right basal bronchus. B. The artificial denture can be seen in the right $B^{9+10}$.

In our study, 1 of 11 patients spontaneously expectorated the foreign body. Chatterji et al. (12) reported that spontaneous expectoration of inhaled foreign bodies occurs in only 1 to 2% cases. Flexible bronchoscopy is useful for the diagnosis and treatment of endobronchial foreign bodies. Of course, the endoscopist should be careful of complications such as asphyxia or massive hemorrhage (13).

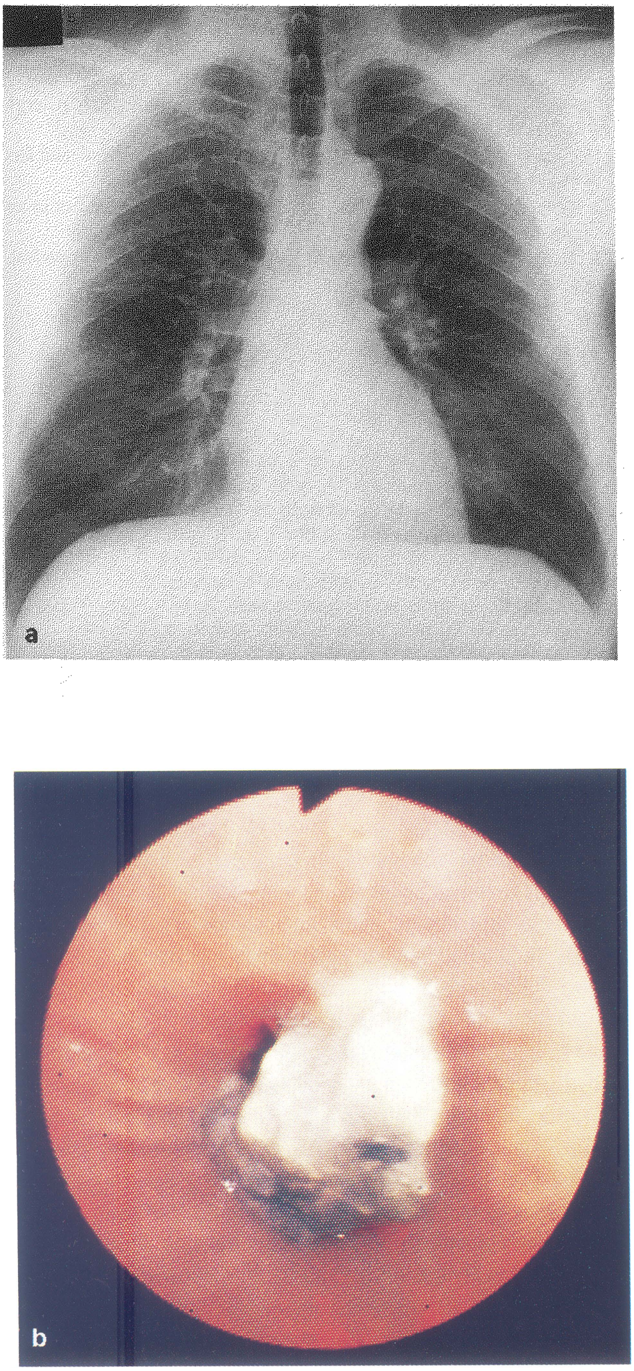

**Figure 3** Case 10. A. Plain chest radiograph 23 days after aspiration of a stone, shows the foreign body in the left main bronchus. B. The stone can be seen in the left main bronchus.

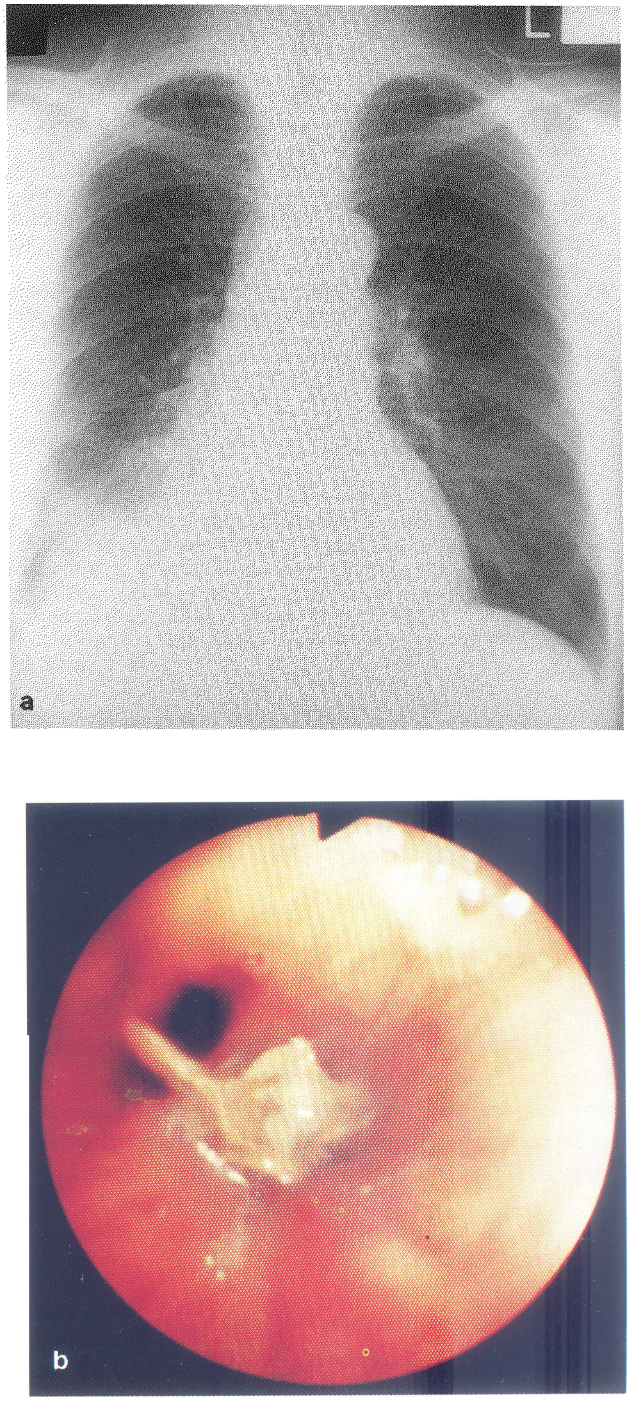

**Figure 4** Case 11. A. Plain chest radiograph on admission shows the atelectasis of the right lower lobe with no evidence of a foreign body. B. Fish bone and granulomatus lesions can be seen in the right lower lobe bronchus.

### REFERENCES

1. Merchant SN, Kirtane MV, et al. Foreign bodies in the bronchi. J Postgrad Med 1984;30:219–223.
2. Katoh T, Andoh M, et al. A case of a bronchial foreign body complicated a pyothorax. J J S B, 1990;12:635–640.
3. Kuwabara M, Itoi K, et al. A case with pneumonia and empyema caused by a bronchial foreign body. J J S B, 1990;12:328–332.
4. Aytaç A, Yurdakul Y, et al. Inhalation of foreign bodies in children. Report of 500 cases. J Thorac Cardiovasc Surg 1977;74:145–151.
5. Rothmann BF, Boeckman C. Foreign bodies in the larynx and tracheobronchial tree in children. A review of 225 cases. Ann Otol 1980;89:434–436.
6. Mantel K, Butenandt I. Tracheobronchial foreign body aspiration in childhood. A report on 224 cases. Eur J Pediatr 1986;145:211–216.
7. Gupta A, Chopra K. Foreign bodies in the tracheobronchial tree. Indian Paediatr 1977;14:133–134.
8. Abdulmajid OA, Ebeid AM, et al. Aspirated foreign bodies in the tracheobronchial tree: Report of 250 cases. Thorax 1976;31:635–640.
9. Campbell DN, Cotton EK, et al. A dual approach to tracheobronchial foreign bodies in children. Surgery 1982;91:178–182.
10. Fine AJ, Abram LE. Asthma and foreign bodies. Ann Allergy 1971;29:217–220.
11. Iwamoto I, Shibata K, et al. A case of a longstanding bronchial foreign body. J J S B, 1989;11:511–514.
12. Chatterji S, Chatterji P. The management of foreign bodies in air passages. Anesthesia 1972;27:390–395.
13. Rees JR. Massive hemoptysis associated with foreign body removal. Chest 1985;88:475–476.

